# Molecular Entanglement Facilitated Improvement of Thermal Stability of Cellulose Diacetate

**DOI:** 10.3390/polym17070835

**Published:** 2025-03-21

**Authors:** Yang Liu, Yin Hu, Jianyu Chen, Zongkai Yan, Lin Zhao, Falu Zhan, Junjie Wang, Yagang Zhang

**Affiliations:** 1 School of Materials and Energy, University of Electronic Science and Technology of China, Chengdu 611731, China; 202221030136@std.uestc.edu.cn (Y.L.); yhu426@uestc.edu.cn (Y.H.); 2023030902008@std.uestc.edu.cn (J.C.); yanzongkai@uestc.edu.cn (Z.Y.); zhaolin316@uestc.edu.cn (L.Z.); 2 The 46th Institute of Sixth Academy CASIC, Hohhot 010010, China; 3 Department of Chemistry, School of Science, Xihua University, Chengdu 610039, China

**Keywords:** cellulose acetate, thermal stability, polyphenylene sulfide, polycarbonate, polyimide, melt extrusion

## Abstract

As a renewable and degradable biomass material, cellulose diacetate (CDA) has significant development potential and has gained widespread interest from researchers. However, its poor thermal stability at high temperatures limits its practical use in the extrusion process and restricts its applications in other fields, such as high-heat airflow filters, battery separators and special textile materials. To enhance the thermal stability of CDA, three heat-resistance additives, i.e., polyphenylene sulfide (PPS), polycarbonate (PC) and polyimide (PI), were introduced to synthesize PPS/CDA, PC/CDA and PI/CDA composite materials through melt extrusion. The incorporation of three heat-resistant additives increased the glass transition temperature (T_g_), initial melting temperature (T_m_^i^) and final melting temperature (T_m_^f^) of the composites, and it reduced the heat loss at 195 °C. After conducting the isothermal thermogravimetry test for 3 h at 215 °C in an air atmosphere, the weight loss of PPS/CDA, PC/CDA and PI/CDA composites was 4.6%, 4.1% and 3.7%, respectively, which was 5.1% lower than that of pure CDA. Morphology characterization tests using a 3D digital microscope and a field emission scanning electron microscope (FESEM) revealed the compatibility order with CDA as the following: PC > PPS > PI. Additionally, Fourier transform infrared spectroscopy (FT–IR) disclosed that hydrogen bonds were formed between heat-resistant additives and CDA molecules, and the carbonyl groups in CDA showed conjugation and hyperconjugation effects with the benzene rings in the additives. Therefore, the enhanced thermal stability of CDA composites can be attributed to the molecular entanglement and crosslinking between additives and CDA molecules.

## 1. Introduction

With growing concern over energy shortages and environmental pollution, renewable and degradable biomass materials have attracted increasing attention [1,2]. Cellulose and its derivatives (ethyl cellulose, hydroxypropyl cellulose, and so on) have been widely used because of their degradability and clean sources [3,4,5]. Cellulose acetate (CA), a derivative of cellulose, has gained widespread interest from researchers because of its high transparency, toughness and easy molding and processing.

CA is a thermoplastic resin produced by the esterification reaction between cellulose and acetic anhydride, which demonstrates many advantages, such as excellent hydrophilicity, low cost, toughness, glossiness, thermoplasticity and biodegradability [6,7,8,9,10,11,12,13]. Based on the degree of hydroxyl substitution (DS) in cellulose molecules by the acetyl group (-COCH_3_), CA can be classified into cellulose monoacetate (CMA), cellulose diacetate (CDA) and cellulose triacetate (CTA), with substitution degrees of 1.72~1.95, 2.22~2.76 and 2.76~3.03, respectively [14,15,16,17,18]. Different DSs endow CA with various properties, broadening its applications across diverse fields, including tobacco, eyeglass frames, pharmaceuticals, plastic products, automobile manufacturing, textiles, etc. [19,20]. Additionally, biodegradability of cellulose acetate depends significantly on its DS. Generally speaking, the lower the DS, the easier biodegradation is [21,22]. With their progress in research and application in high-end fields such as liquid crystal displays, biosensors and energy storage materials, CAs have played an increasingly important role in these fields as environmentally friendly and renewable materials [23,24].

Despite the numerous advantages of CA, its poor thermal stability at high temperatures presents a significant challenge in practical use. For example, the production of CA colloidal particles often involves a melt extrusion of the raw material, which imposes high requirements on the thermal stability of CA. In addition, the low thermal stability of CA also limits its application in areas such as high-heat airflow filters and battery separators [25,26]. Therefore, improving the heat resistance of CA holds significant practical importance.

In general, enhancing the thermal stability of CA relies on two main strategies: the incorporation of CA with inorganic particles or polymer materials and the chemical modification of CA. The mechanism underlying these strategies primarily involves the introduction of heat-resistant additives (e.g., inorganic particles, polymers, organic compounds) or function groups, along with the interactions formed between additives or function groups and CA [27,28,29,30]. For example, by using CA and calcium oxide (CaO) as raw materials, the resulting CA–CaO separator shows higher thermal stability than neat CA. When CaO was added to form CA–CaO, the temperature of decomposition (approximately 320 °C) was increased by approximately 60 °C compared to that of neat CA (about 260 °C). The enhancement of thermal stability can be attributed to the high melting point of CaO and the complexation between CaO and the carbonyl group in CA [26]. Woo-Il Baek et al. prepared cellulose acetate/poly (butyl acrylate) (CA/PBA) composite fibrous mats by an electrospinning method with PBA as an adhesive and CA as a matrix. By increasing the amount of PBA in the hybrid mats, a gradual increase in the thermal degradation temperature of CA/PBA could be observed (The observed maximum degradation temperatures for the first peak were 122.8, 128 and 147 °C for 10%, 20% and 30% PBA, respectively), which could be attributed to the interaction of the two polymers through point-bonded structures [31]. Meanwhile, modification of CA with 4-nitro (phenyl amino) maleimide can also significantly increase the initial decomposition temperature of CA (an increase of 50 °C), as the functional groups are thermally stable and can form intramolecular hydrogen bonds between the nitro groups and the hydroxyl groups [25].

**Figure 1:**
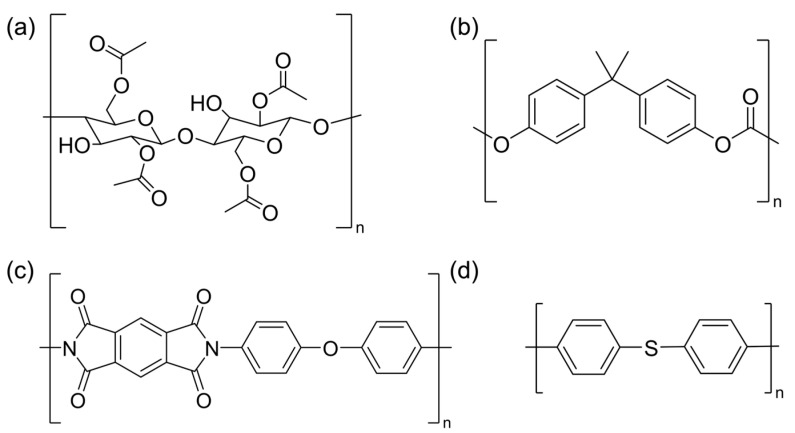
Molecular structural formulas of CDA (**a**), PC (**b**), PI (**c**) and PPS (**d**).

Compared with chemical modification, the incorporation of CA with additives exhibits advantages in the simplicity of the process and the low cost in industrial production. Polyphenylene sulfide (PPS, (C_12_H_8_S)_n_), polycarbonate (PC, (C_16_H_14_O_3_)_n_) and polyimide (PI, (C_22_H_10_O_5_N_2_)_n_) are high-performance materials renowned for their excellent heat and flame resistance, high mechanical strength and chemical stability [32,33,34]. As a result, they are widely applied in aviation, aerospace, microelectronics, liquid crystals, separation membranes, etc. Owing to the aromatic rings in their polymer structures, PPS, PC and PI demonstrate strong thermal stability, making them ideal additive candidates for enhancing the thermal stability of CA. The chemical structures of CDA, PPS, PC and PI are shown in Figure 1. So far, no research has been reported to explore PPS, PC and PI as additives mixed with CDA. This is the first time these additives have been explored.

Herein, we employed PPS, PC, and PI as heat-resistant additives, to synthesize three composites of CDA materials through melting extrusion, namely, PPS/CDA, PC/CDA, and PI/CDA composite materials. As plasticizers and antioxidants are crucial for reducing the melt viscosity, enhancing the product flexibility and preventing oxidation and thermal degradation during melt extrusion process, the environmentally friendly citrate plasticizer triethyl citrate (TEC), along with antioxidants tetrakis [methylene(3,5-di-tert-butyl-4-hydroxyhydrocinnamate)]methane (Irganox 1010) and tris(2,4-di-tert-butylphenyl)phosphite (Irgafos 168), were introduced to ensure the smooth processing and maintain the material properties [19]. The thermal properties of CDA composites were characterized by differential scanning calorimetry (DSC), melting point apparatus test and thermogravimetric (TG) analysis, while the interaction between CDA and additives were investigated using dynamic mechanical analysis (DMA), 3D digital microscope, field emission scanning electron microscope (FESEM), and Fourier transform infrared spectroscopy (FT–IR).

## 2. Materials and Methods

### 2.1. Materials

Cellulose diacetate (CDA) powder (CS-Grade) with a DS of 2.42 and free from additives, was supplied by Sichuan Push Acetati Co., Ltd. (Yibin, China). The antioxidants Irganox1010 and Irgafos168 were purchased from BASF GmbH (Ludwigshafen, Germany). Triethyl citrate (TEC) was procured from Beijing Bailingwei Technology Co., Ltd. (Beijing, China). Polyphenylene sulfide (PPS, (C_12_H_8_S)_n_), polycarbonate (PC, (C_16_H_14_O_3_)_n_) and polyimide (PI, (C_22_H_10_O_5_N_2_)_n_) were obtained from Dongguan Xinmiao New Material Co., Ltd. (Dongguan, China), Covestro (Leverkusen, Germany) and Dongguan Zhan Yang Polymer Materials Co., Ltd. (Dongguan, China), respectively.

Molecular weight information acquired from the GPC test of CDA, PPS, PC and PI is shown in Table 1. The GPC test was conducted with an Agilent PL-GPC50 (Santa Clara, CA, USA). The solvents used in the GPC test were a-Clonai for PPS and tetrahydrofuran for CDA, PC and PI. The chemical structures of CDA, PPS, PC and PI are shown in Figure 1.

### 2.2. Preparation of CDA Composites

The formulations of the blends studied in this work are summarized in Table 2. Based on the formulations presented in Table 2, the corresponding mass ratio of ingredients were weighed, mixed and stirred, and preheated (100 °C) to ensure homogeneity before extrusion. Then, they were extruded using a double-screw extruder (CT30/15, Jiangsu Orode Machinery Co., Ltd., Suzhou, China) at a speed of 50 rpm and an extrusion temperature of 195 °C. Pure CDA was extruded at 215 °C, while the PPS, PC and PI powder blends were not extruded, but directly subjected to further testing. The resulting extrudates were cooled, cut into pieces, and stored in a dry environment.

### 2.3. Characterization of CDA-Based Materials

#### 2.3.1. Thermogravimetric Analysis

TG analysis was performed using a NETZSCH STA 449F5 (Selb, Germany). Samples from each formulation were heated at a rate of 10 °C·min^−1^ from 25 °C to 800 °C under a nitrogen flow of 20 mL·min^−1^. Isothermal TG analysis was performed using a NETZCH TG 209F1 Libra at 215 °C under an air flow of 20 mL·min^−1^.

#### 2.3.2. Differential Scanning Calorimetry

The glass transition temperatures (T_g_) were measured by DSC using a NETZSCH DSC 214 (Selb, Germany) under constant nitrogen flow. All samples (less than 10 mg) were first heated to 250 °C, then cooled to 25 °C at a heating and cooling rate of 10 °C·min^−1^ to eliminate their thermal history. Subsequently, they were reheated to 250 °C at the same heating rate, and the second heating scan was recorded. T_g_ was defined as the temperature corresponding to the point at the maximum slope in the DSC curve.

#### 2.3.3. Melting Point Measurement

Initial melting temperatures (T_m_^i^) and final melting temperatures (T_m_^f^) were measured using a Digipol-M80 (Jiahang Instruments, Shanghai, China). Samples were heated at a heating rate of 20 °C·min^−1^ until completely melted, with the entire process recorded on video. T_m_^i^ was defined as the temperature at which melting began, while T_m_^f^ was the temperature at which the sample was completely melted into liquid.

#### 2.3.4. Dynamic Mechanical Analysis

DMA measurements were carried out by a DMA850 (Waters Corporation, Milford, CT, USA). CDA-based materials were first hot-pressed into cubic strips at 200 °C using a small, flat vulcanizer. Sample strips had dimensions of 40 mm (length), 8 mm (width) and 2 mm (thickness). DMA measurements were conducted in three-point bending mode with a frequency of 1 Hz and a heating rate of 2 °C·min^−1^.

#### 2.3.5. 3D Digital Microscopy

3D digital microscopy images were captured by a KS-X1000 (Nanjing Kathmatic Technology Co., Nanjing, China).

#### 2.3.6. Field Emission Scanning Electron Microscopy

The cross sections of CDA-based materials were observed on an Ultim Max40 FESEM instrument (Oxford Instruments, Oxford, UK). Sample strips were prepared as described in the DMA section and then freeze-fractured in liquid nitrogen. The fracture surfaces were vacuum coated with gold and examined at an acceleration voltage of 10 KV.

#### 2.3.7. Fourier-Transform Infrared Spectroscopy

FT–IR measurements were performed in attenuated total reflection (ATR) mode using an Invenior spectrometer (Bruker Co., Billerica, MA, USA). Sample strips were prepared as depicted in DMA measurements. The spectra were recorded in the frequency range of 400–4000 cm^−1^.

## 3. Results and Discussion

### 3.1. Thermal Properties

**Figure 2:**
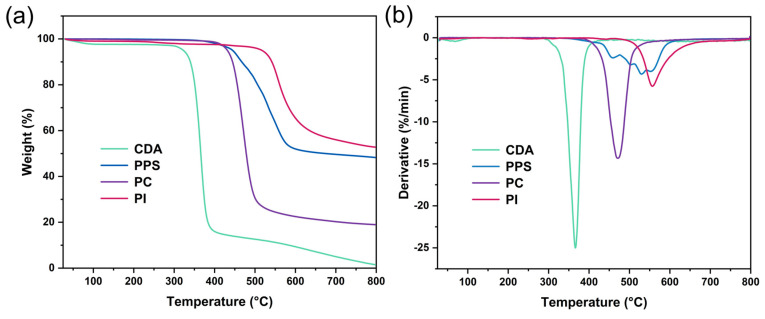
TG (**a**) and DTG (**b**) curves for CDA, PPS, PC and PI.

The heat resistance of the four raw materials used in this study (i.e., CDA, PPS, PC and PI) was evaluated first. Figure 2 shows the TG and derivative thermogravimetric (DTG) curves for these materials. CDA exhibited the lowest heat resistance among the four materials, with the lowest degradation temperature and the greatest weight loss across all temperature ranges. The thermal degradation of PPS and PC occurred at higher temperatures compared to CDA, indicating superior intrinsic heat resistance. Additionally, PI demonstrated excellent thermal stability with a degradation peak above 550 °C, making it the most thermally stable material among the four despite a slight weight loss below 500 degrees (Figure 2, Table 3).

**Figure 3:**
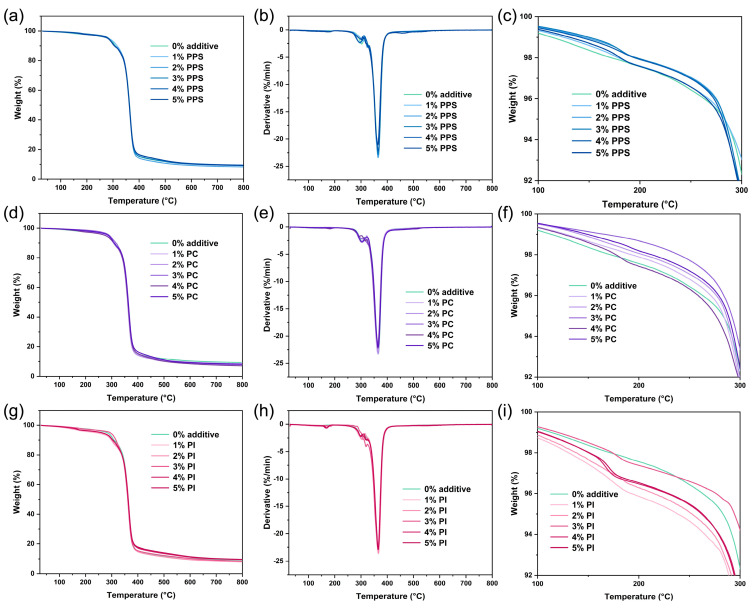
TG curves (**a**,**d**,**g**), DTG curves (**b**,**e**,**h**) and partial curves of TG (100–300 °C) (**c**,**f**,**i**) for different CDA-based samples.

**Figure 4:**
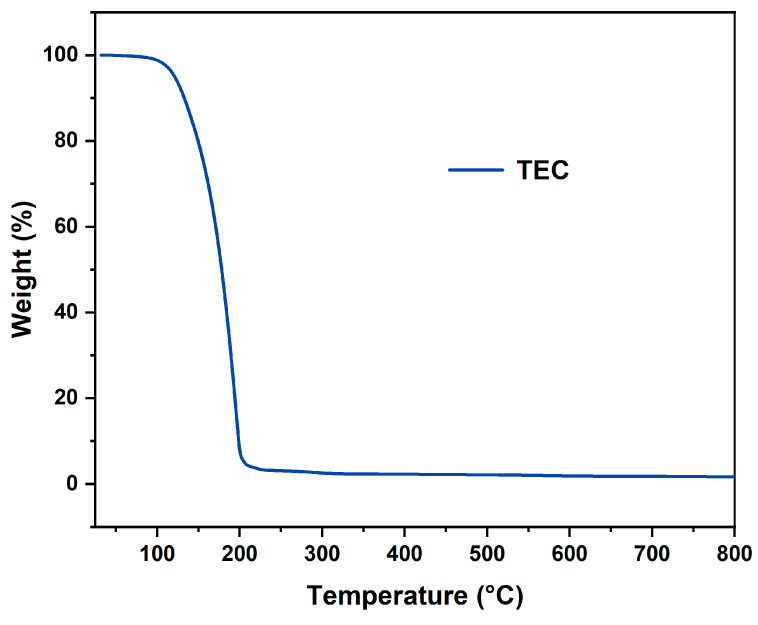
TG curves for TEC.

TG analysis was subsequently performed to evaluate the thermal decomposition behavior and heat loss of different CDA-based samples. Figure 3 displays TG curves, DTG curves and magnified TG curves within the 100–300 °C range for these samples. The observed weight loss between 150 and 340 °C was ascribed to the volatilization of the TEC plasticizer, resulting in a slight loss in mass (Figure 4) [19]. The maximum degradation rates for all samples appeared at approximately 365 °C (Table 3), primarily attributed to the cleavage of glycosidic bonds in cellulose backbones, dehydration, depolymerization and the loss of acetate groups [35,36]. The final stage of weight loss occurred between 365 °C and 800 °C, corresponding to the complete decomposition and carbonization of the CDA [16].

The melting processing temperature range of CDA lies between 150–250 °C. To distinguish differences in thermal stability within this processing temperature range, TG curves were truncated and magnified for the 100–300 °C interval (Figure 3c,f,i). As illustrated in Figure 4 and Table 3, the residual mass at 195 °C (Mass_195°C_) of CDA composites increased significantly with the introduction of PPS, which was attributed to the inherent excellent heat resistance of PPS. Compared to PPS, PC had a more pronounced effect in increasing Mass_195 °C_ as a heat-resistant additive (except for 4% PC). Interestingly, despite the superior intrinsic thermal stability of PI, the addition of PI reduced the Mass_195°C_ of CDA composites. PI’s stability is evident from the thermogravimetric analysis (TG), which showed approximately 10% residue at 800 °C—slightly higher than the other composites.

The T_g_ of raw materials and CDA composites with varying contents of PPS, PC and PI were determined using DSC.

**Figure 5:**
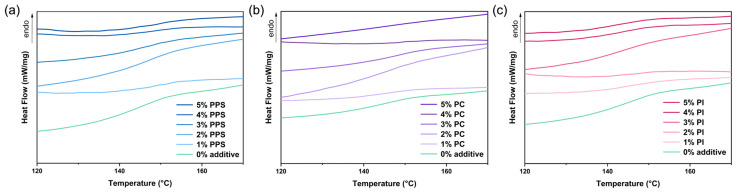
DSC thermograms of different CDA composites with varying contents of PPS (**a**), PC (**b**) and PI (**c**).

**Figure 6:**
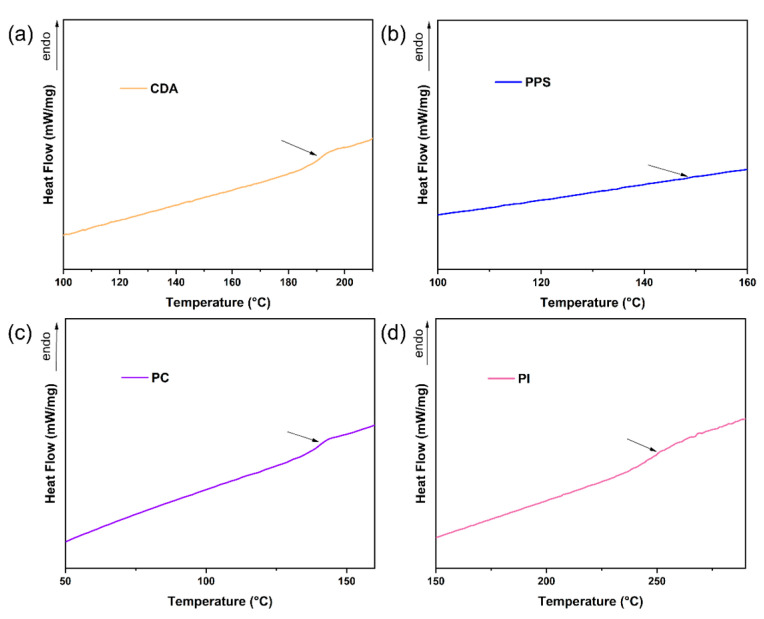
DSC thermograms of raw materials, CDA (**a**), PPS (**b**), PC (**c**) and PI (**d**).

The DSC thermograms are presented in Figure 5 and Figure 6, and the corresponding T_g_ values are listed in Table 3. According to the chain relaxation theory, when the temperature reaches the glass transition temperature, the polymer chain segment changes from a glassy state to a high-elastic state, which means that the chain segment begins to move freely. With the addition of PPS, PC and PI, the T_g_ of CDA composites shifted to higher temperatures (e.g., 150.2 °C for 5% PPS, 149.5 °C for 3% PC and 150.3 °C for 1% PI, compared to 145.2 °C for no additive). The results demonstrated that the chain segment is more difficult to move with the right shift of point at the maximum slope in the DSC curves. Similarly, the introduction of three heat-resistant additives also resulted in an increase in T_m_^i^ and T_m_^f^ at varying degrees (Table 3), which widened the service temperature range and raised the upper thermal limit of CA composites.

The additives of 2% PPS, 3% PC and 3% PI were selected as optimum samples to perform the following measurements and characterization for their least Loss195 °C within respective groups. Despite 2% PPS, 3% PPS and 4% PPS in this series having similar weight loss, 2% PPS was selected as the optimum sample for achieving the best modification result with the least amount of PPS, thus having the lowest cost of raw materials.

**Figure 7:**
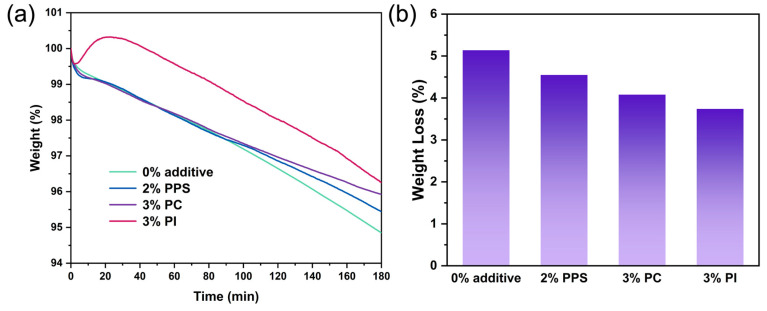
Isothermal TG curves (**a**) and final weight loss (**b**) for 3 h at 215 °C in an air atmosphere of CDA composite with 0% additive, 2% PPS, 3% PC and 3% PI.

To simulate the practical extrusion conditions involving prolonged exposure to high temperatures, three hours of uninterrupted thermogravimetric tests were conducted at 215 °C in air atmosphere. As shown in Figure 6a, the weight loss rates of CDA composites with 2% PPS and 3% PC were slower than that of CDA without an additive. The rapid initial weight loss in the first 10 min across all samples could be attributed to the volatilization of small molecule plasticizers. Interestingly, the weight of the CDA composite with 3% PI additive increased significantly during the early stage of the isothermal TG test, even surpassing its initial weight, possibly due to the absorption of oxygen and other substances from the air and subsequent chemical reactions [37]. When cellulose diacetate is heated, it releases acetic acid groups (CH_3_COOH), which may catalyze the oxidation of PI, thus leading to the absorption of oxygen and an increase in mass [38]. After 3 h, the final weight loss measurements for CDA composites with 0% additive, 2% PPS, 3% PC and 3% PI were 5.1%, 4.6%, 4.1% and 3.7%, respectively (Figure 7b). These results suggest that PPS/PC/PI-modified CDA composites exhibited reduced weight loss during extrusion. Although 3% PI showed the least final weight loss in four samples, it might also have undergone a change in chemical composition [39].

**Figure 8:**
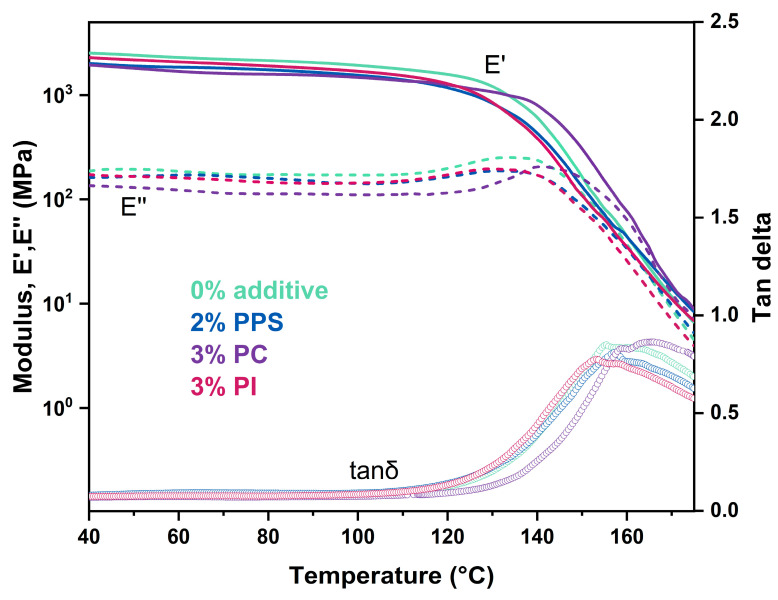
E′ (solid line), E″ (dash line) and tanδ (dotted line) of CDA composites with 0% additive, 2% PPS, 3% PC and 3% PI, obtained by DMA measurement.

DMA was performed to investigate the molecular structural changes of CDA composites under dynamic temperature. Figure 8 exhibits the temperature dependence of storage modulus (E′), loss modulus (E″) and loss tangent (tanδ) for CDA composites with different contents of heat-resistant additives. The storage modulus represents the energy stored by the elastic deformation of the viscoelastic material during deformation, and the loss modulus describes the energy lost by the material during deformation. Tanδ is defined as the ratio of E″ to E′. Below 130 °C, all modified samples displayed decreased E′ compared to unmodified CDA. As the temperature continued to rise, the E′ of the CDA composite with 3% PC decreased the slowest, revealing it as having the best heat resistance. Since E′ reflects material stiffness, its decrease suggests enhanced flexibility in modified CDA composites. The reduction of E′ at lower temperatures is due to diminished hydrogen bonds between CDA and TEC molecules, thus promoting the segmental mobility in the CDA backbone. The tanδ peak corresponded to the T_g_ values of the CDA composites with 0% additive, 2% PPS, 3% PC and 3% PI, which, as measured by DMA, were 155.4 °C, 157.1 °C, 158.9 °C and 153.2 °C, respectively. The variation in T_g_ values between DMA and DSC methods (vide supra) mainly arises from the differences in relaxation process and data evaluation approaches in the two techniques [40].

### 3.2. Morphology and Composition Characterization

**Figure 9:**
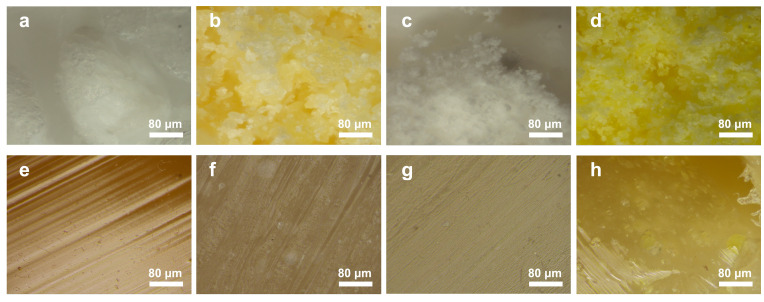
3D digital microscope images of CDA (**a**), PPS (**b**), PC (**c**), PI (**d**) and CDA composites with 0% additive (**e**), 2% PPS (**f**), 3% PC (**g**) and 3% PI (**h**).

Using a 3D digital microscope, the micromorphology of raw materials and modified samples was observed. Figure 9 presents the morphology of the four raw materials and the four extruded samples. The raw materials exhibited varying particle size distribution and color appearance (Figure 9a–d). For example, CDA and PC powders were white, while PPS and PI powders were yellow (Figure 9a,c vs. Figure 9b,d). Despite imperfections like scratches on the surface and tiny bubbles within the matrix, all raw materials were uniformly extruded. As for the CDA composites, no significant phase separation was observed in composites with 2% PPS and 3% PC, demonstrating effective compatibility between PPS/PC and CDA. However, independent PI particles larger than 50 μm were observed embedded in the CDA matrix (Figure 9h), exhibiting a phase separation structure identified as a typical sea (CDA)-island (PI) morphology [41].

**Figure 10:**
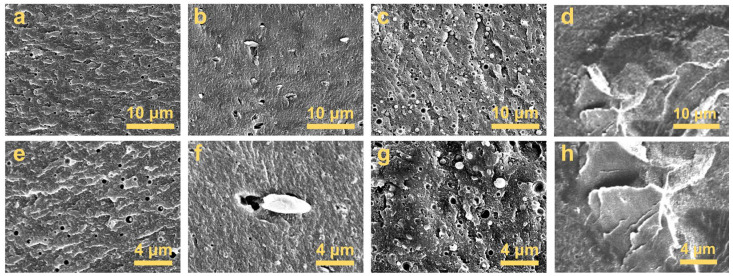
FESEM images of freeze-fractured surface morphologies of CDA composites with 0% additive (**a**,**e**), 2% PPS (**b**,**f**), 3% PC (**c**,**g**) and 3% PI (**d**,**h**).

**Figure 11:**
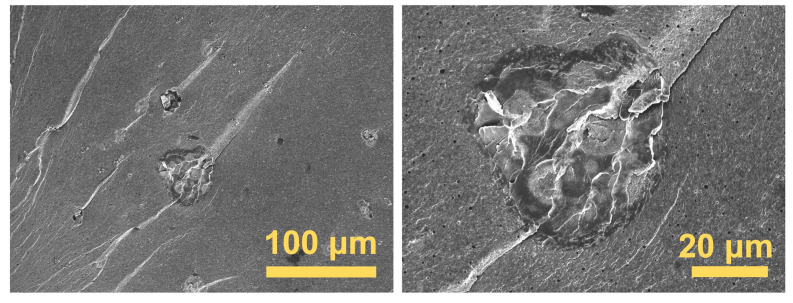
FESEM images of freeze-fractured surface morphologies of CDA composites with 3% PI.

In order to further explore the compatibility differences between various additives and CDA, freeze-fractured surface morphologies were analyzed using a FESEM (Figure 10 and Figure 11). Tiny bubbles less than 1 μm in diameter were observed within the composites’ matrices due to the volatilization and escape of TEC plasticizers during extrusion [19]. At higher magnifications, independent PPS particles were seen adhering to the CDA matrix (Figure 10b,f). Compared to PPS, PC was more evenly dispersed in the matrix, forming more rounded and smaller globules (Figure 10c,g). In contrast, PI particles with diameters exceeding 50 μm were distinctly larger than PC and PPS (Figure 10d,h and Figure 11), which was consistent within other observations from the 3D digital microscope (Figure 9h).

When combining the results from the 3D digital microscope and FESEM images, PC exhibited the best compatibility with CDA, followed by PPS, while PI showed the poorest compatibility. As listed in Table 3, PPS and PC had relatively low T_g_ and T_m_^i^ and thus were molten or physically fragmented during the high-temperature extrusion process, resulting in their effective compatibility with CDA [42,43,44]. Conversely, the T_g_ of PI (>195 °C) prevented its complete melting during extrusion, thus contributing to its large particle sizes and poor incorporation with CDA [45]. The poor compatibility of PI with CDA might be the reason for its lower effect on enhancing the thermal stability of CDA; larger particles destroyed the original structure of CDA to some extent, leading to the unusual behavior observed around 200 °C in the PI composite and unexpectedly low T_g_ values for CDA–PI composites, as shown in Table 3.

**Figure 12:**
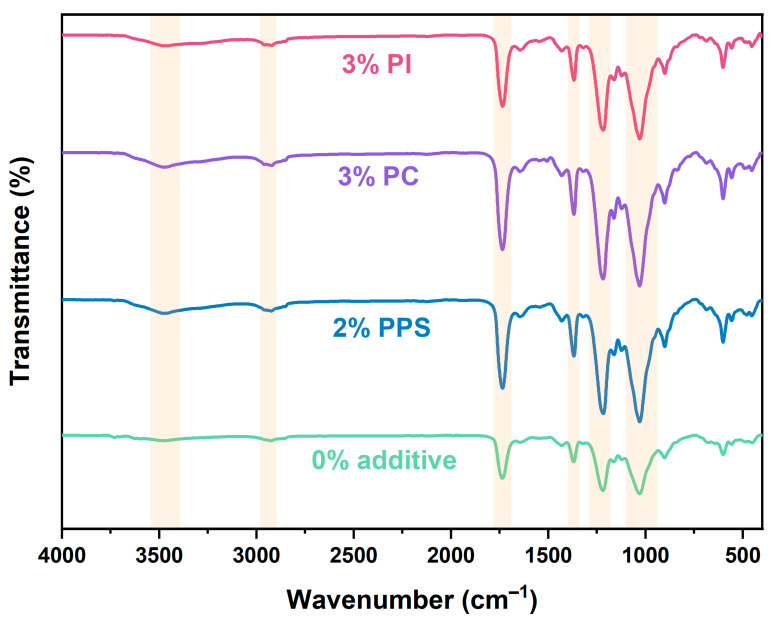
The FT–IR spectra of CDA composites with 0% additive, 2% PPS, 3% PC and 3% PI.

The chemical interactions of additives with CDA were investigated by an FT–IR spectral analysis of the blends. Figure 12 shows the FT–IR spectra for CDA composites with 0% additive, 2% PPS, 3% PC and 3% PI, with the corresponding peak assignments listed in Table 4. In the 3400–3500 cm^−1^ range, the broad peaks assigned to O-H stretching vibrations shifted from 3478 cm^−1^ (without additive) to about 3475 cm^−1^ (with additive) [40]. This shift indicated the formation of hydrogen bonds, as hydrogen bonding affects the vibration energy levels and typically results in a lower O-H stretching frequency. The characteristic peak at 1219 cm^−1^ is attributed to the C-O bond stretching of the C-O-H group in CA [16]. The peak shifted to lower wavenumbers upon the addition of additives, further confirming the generation of hydrogen bonds between the additives and CDA molecules and the reduction of intermolecular and intramolecular hydrogen bonds of CDA molecules [40,46]. The broad peak at 2925 cm^−1^ was attributed to C-H aromatic vibrations of CDA [12]. Another band at 1736 cm^−1^ was associated with the stretch vibrational modes of the carbonyl ester (C=O). Characteristic bands for CDA were found at 1030 cm^−1^ and at 1370 cm^−1^, which may be associated with the vibrational modes of the C-O-C binding in the anhydroglucose ring of CDA and with the stretching vibrational modes of the C-H binding of -CH_3_ groups present in CDA radicals [19,31,35,41,47]. As illustrated in Figure 1, all three additives contained benzene ring structures. The shift to lower wavenumbers for C=O bonds caused by the introduction of additives indicated the π-π conjugation effect between C=O bonds and benzene rings. Moreover, the σ-π hyperconjugation effect between C-H and -CH_3_ with benzene ring structures further reduced the corresponding wavenumbers (2925 cm^−1^ for C-H and 1370 cm^−1^ for -CH_3_) [48,49]. All these results collectively indicated that three heat-resistant additives could form strong interactions and be tightly entangled with CDA molecules, thus enhancing the thermal stability of CDA composites.

**Figure 13:**
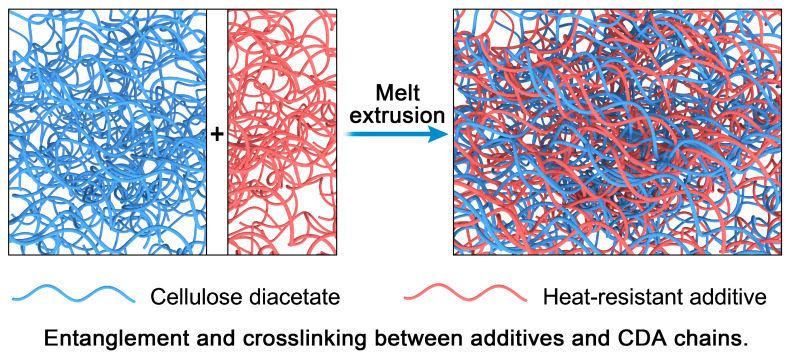
Mechanism of improving thermal stability of CDA by the introduction of high thermal stability additives.

The results of FESEM and FT–IR indicated that at the point of contact between CDA and additives, the two molecules are tightly attached. The tight binding of additives with high heat resistance with the CDA molecules at the point of contact produced a crosslinked structure between additives and CDA during the melt extrusion, which facilitated the formation of hydrogen bonds and the conjugation/hyperconjugation effect. We proposed a hypothetical model, as shown in Figure 13. As a result, the thermal stability of the generated composites was significantly improved. This provides meaningful guidance for the selection and development of heat-resistant modification additives for CDA materials. Heat-resistant additives of different concentrations crosslinked with cellulose diacetate molecules at high temperatures in the extruder, forming a tight entanglement network and improving the heat resistance of cellulose diacetate materials, as shown in Figure 13.

The additives used in this study (PPS, PC and PI) are non-biodegradable. This raises concerns about whether their inclusion compromises the biodegradable and sustainable objectives of the material. In future research on enhancing the thermal stability of cellulose acetate, alternative carbon-based additives, such as nanodiamonds or nanotubes, which may enhance mechanical and thermal properties while aligning better with sustainability goals, might be used and further studied [20,27,28].

## 4. Conclusions

Three thermally stable raw materials, i.e., PPS, PC and PI, were selected as heat-resistant additives and blended with CDA, TEC plasticizer, and Irganox1010 and Irgafos168 antioxidants to synthesize PPS/CDA, PC/CDA and PI/CDA composite materials through melt extrusion. Thermal analysis, including DSC and TG, revealed that the thermal stability of PPS-, PC- or PI-modified CDA composite materials was significantly improved compared to unmodified CDA composites. The addition of three heat-resistant additives increased the T_g_, T_m_^i^ and T_m_^f^ of the composites, as well as reducing the heat loss at 195 °C. After the isothermal thermogravimetry test for 3 h at 215 °C in air an atmosphere, the weight loss values of the PPS/CDA, PC/CDA and PI/CDA composites were 4.6%, 4.1% and 3.7%, respectively, which was lower than that of unmodified CDA composites (5.1%). DMA analysis demonstrated that the addition of three heat-resistant additives reduced the E′ of CDA composites at lower temperatures. Characterization using a 3D digital microscope, FESEM and FT–IR revealed that the compatibility order of the additives with CDA was PC > PPS > PI. The entanglement and crosslinking between additives and CDA molecules resulted in the formation of hydrogen bonds and the creation of conjugating and hyperconjugating effects between function groups, thus enhancing the thermal stability of CDA composites. To conclude, a novel heat-resistant modification strategy for CDA was developed using materials with low glass transition temperatures and melting temperature, but high thermal decomposition temperature as heat-resistant additives for co-melt extrusion. This study is expected to provide valuable guidance for the selection and development of heat-resistant modification additives for CDA.

## Figures and Tables

**Table 1:** Molecular weight information acquired from GPC test for several raw materials.

Material	Mn (kDa)	M_w_ (kDa)	M_p_ (kDa)	PDI
CDA	42.7	194	151	4.54
PPS	18.7	44.4	43.0	2.37
PC	17.6	52.3	46.0	2.97
PI	21.0	32.1	27.0	1.53

**Table 2:** Materials and components of heat-resistant modified CDA-based samples.

Materials	CDA (wt, %)	TEC (wt, %)	Irganox 1010 (wt, %)	Irgafos 168 (wt, %)	PPS (wt, %)	PC (wt, %)	PI (wt, %)
CDA	100	/	/	/	/	/	/
PPS	/	/	/	/	100	/	/
PC	/	/	/	/	/	100	/
PI	/	/	/	/	/	/	100
0% additive	89	10	0.5	0.5	/	/	/
1% PPS	88	10	0.5	0.5	1	/	/
2% PPS	87	10	0.5	0.5	2	/	/
3% PPS	86	10	0.5	0.5	3	/	/
4% PPS	85	10	0.5	0.5	4	/	/
5% PPS	84	10	0.5	0.5	5	/	/
1% PC	88	10	0.5	0.5	/	1	/
2% PC	87	10	0.5	0.5	/	2	/
3% PC	86	10	0.5	0.5	/	3	/
4% PC	85	10	0.5	0.5	/	4	/
5% PC	84	10	0.5	0.5	/	5	/
1% PI	88	10	0.5	0.5	/	/	1
2% PI	87	10	0.5	0.5	/	/	2
3% PI	86	10	0.5	0.5	/	/	3
4% PI	85	10	0.5	0.5	/	/	4
5% PI	84	10	0.5	0.5	/	/	5

**Table 3:** Thermal properties of raw materials and CDA composites *.

Formulations	T_g_ (°C)	T_m_^i^ (°C)	T_m_^f^ (°C)	Mass_195°C_ (%)	T_dmax_ (°C)
CDA	191.5	205.7	246.4	97.6	365.9
PPS	148.7	231.1	261.2	99.9	529.4
PC	140.1	185.9	234.9	99.6	471.3
PI	250.9	274.6	323.0	98.9	557.3
0% additive	145.2	211.6	261.7	97.6	366.1
1% PPS	148.3	214.5	264.2	97.7	365.4
2% PPS	148.2	217.9	268.7	98.0	365.1
3% PPS	149.1	214.8	266.8	98.0	365.1
4% PPS	149.3	213.8	277.7	98.0	365.5
5% PPS	150.2	213.7	260.6	98.0	364.1
1% PC	147.1	216.2	263.6	97.8	363.2
2% PC	147.7	218.9	270.7	98.1	365.2
3% PC	149.5	224.6	274.4	98.7	363.3
4% PC	149.3	226.6	274.9	97.5	364.4
5% PC	148.9	227.2	275.1	98.3	365.2
1% PI	150.3	214.7	263.4	96.0	364.7
2% PI	149.4	215.3	264.9	96.4	365.9
3% PI	147.1	216.2	265.6	97.4	364.1
4% PI	147.2	214.7	265.4	96.5	365.4
5% PI	145.3	214.4	264.9	96.6	365.7

* T_g_ was derived from DSC curves. T_m_^i^ and T_m_^f^ were measured by the melting point apparatus. Mass_195 °C_ and T_dmax_, representing residual mass at 195 °C and the temperature at maximum weight loss rate, respectively, were acquired from results of TG.

**Table 4:** Peak assignments of FT–IR spectra.

Formulations	Wavenumber (cm^−1^)					
	O-H Stretching Vibration	C-H Stretching Vibration	C=O Stretching Vibration	CH_3_ Bending Vibration	C-O-H Stretching Vibration	C-O-C Stretching Vibration
0% additive	3478	2925	1736	1370	1219	1030
2% PPS	3475	2922	1735	1368	1217	1030
3% PC	3475	2921	1735	1368	1218	1030
3% PI	3474	2921	1735	1368	1217	1030

## Data Availability

The data presented in this study are available upon request from the corresponding author.
